# Editorial: Quality of life in academia: new perspectives for assessing and promoting wellbeing in university population

**DOI:** 10.3389/fpsyg.2024.1515372

**Published:** 2025-01-07

**Authors:** Barbara Loera, Andreina Bruno, Sara Viotti, Imke Hindrichs

**Affiliations:** ^1^Department of Psychology, University of Turin, Turin, Italy; ^2^Department of Education Sciences, University of Genoa, Genoa, Italy; ^3^Centro de Investigación Transdisciplinaria en Psicología, Universidad Autónoma del Estado de Morelos, Cuernavaca, Mexico

**Keywords:** higher education, quality of life, work related stress, academic stress, health measurement, wellbeing, implementation gap, academia

In recent decades, significant changes have transformed academic organizations, reshaping the objectives, roles, and social image of university staff. The growing emphasis on performance accountability, now extending to public universities, has intensified the focus on internationalization, competition, fundraising, and the managerial aspects of academic work. Professors and researchers are now expected to excel not only in teaching and research but also in securing funding, patenting research outcomes, transferring knowledge to local contexts, engaging in public outreach, and fulfilling institutional responsibilities.

These changes have also impacted technical and administrative staff, who are responsible for implementing various projects and accountability policies. Students have also been affected by these shifts, and although many changes have been designed to improve their education and employability, it is undeniable that they now face increased demands for efficiency and adaptability in an often uncertain and complex labor market.

In essence, today's academic organizations are highly dynamic and challenging environments. While motivating and rewarding, they are becoming increasingly demanding and pose significant risks to psychological wellbeing and, more broadly, the quality of academic life. The COVID-19 pandemic has introduced additional challenges, further increasing the psychosocial demands of academic work and study.

Research on the quality of life in academia has often borrowed from the broader occupational health literature, without fully addressing the unique complexities of the evolving academic context. Existing policies and interventions also fall short in considering contemporary factors influencing wellbeing in universities.

There is a growing awareness and interest among academics in how these risks can be addressed or, better still, prevented in this unique professional and educational setting.

Various working groups and initiatives across Europe, such as Healthy Universities (https://healthyuniversities.ac.uk/), the ARK Intervention Programme (https://www.ntnu.edu/ark/the-ark-intervention-programme), REMO (Research Mental Health Observatory, https://projects.tib.eu/remo/), and the QoL@W network (Quality of Life at Work in academia, https://aipass.org/gruppi-tematici/qolwork-quality-of-life-at-work/) in Italy aim to foster a culture of wellbeing in academia.

These initiatives show that it is crucial to better assess the quality of life in academia, to facilitate comparative studies, and to inform the development of appropriate policies and interventions that promote the wellbeing of the university population.

The Research Topic on the Quality of Life in Academia represents a valuable contribution in this desirable direction, accounting for the specificities of the academic organizational context as well as the unique characteristics of the population involved in Europe, Asia, and Latin America.

The articles in this Research Topic reflect the current focus on wellbeing in academia, with much attention paid to students. Of the 12 accepted articles, six are dedicated to students at different educational levels, from undergraduates to doctoral students.

Two papers focus on assessing the psychometric properties of tools used to evaluate various aspects of students' wellbeing. Menardo et al. introduce the Perceived Restorativeness Scale (Rest@US), confirming the importance of university physical environments in enhancing student wellbeing. Matavovszky et al. aim to validate the Outcome Questionnaire, useful for assessing mental health, in a sample of Hungarian university students, using a toolkit that includes measures of depression, resilience, social support, and burnout.

Quiroga-Castañeda et al. examine the potential long-term health consequences for students reporting stress, particularly in relation to the irritable bowel syndrome. Their study of 403 Peruvian medical students highlights the need for preventive measures against academic stress and its adverse effects on physical health.

The qualitative study by Mikhaylova et al. explores students' life choices and finds that educational decisions are considered by Russian university students to be the most crucial in shaping their future. This study suggests the importance of vocational guidance programs to support students' wellbeing and improve quality of life in academia.

Two further contributions focus on a specific group: doctoral students and postdoctoral researchers. These individuals are in a transitional phase between students and academic workers, dedicating much of their time to training and learning while also taking on significant research and teaching responsibilities. These studies, conducted by Vilser et al. in Germany and Bacci et al. in Italy, explore the challenges faced by this group, including job insecurity, effort-reward imbalance, and workload, and how these factors impair their quality of life.

Overall, while it is commendable that researchers and professors are concerned about the wellbeing of their students, it is also essential to place greater emphasis on the quality of working life for other university populations: professors, researchers, and technical-administrative staff. This is the focus of the remaining articles.

Starting with professors, psychological malaise in teaching is demonstrated by Pei et al., who show that intentions to leave among Chinese university professors, especially those with low self-efficacy, are intensified under stress.

The impact of the coronavirus on academic life is given special attention. Díaz et al. examine the psychosocial factors affecting both professors and students working or studying from home during the COVID-19 lockdown in Mexico. They found that students were more vulnerable, highlighting not only the pandemic-related factors, but also issues relevant to remote working and studying in general.

Capone et al. also emphasize the importance of learning from remote working during the pandemic and integrating these lessons into the post-pandemic “new normal” in Italian universities. They also focus on technical-administrative staff (TAS), a frequently overlooked group and stress the importance of social support for this category of workers.

Bruno, Buono et al. address this group as well by validating the “Technical and Administrative Staff Quality of Life At Work” (TASQ@work) tool, developed by the QoL@Work, the Italian network of academic Work and Organizational Psychologists. The tool combines various measures to assess TAS quality of life, in order to inform better organizational policies.

Attention to the social dimension is also present in the study by Signore et al. in Southern Italy, which emphasizes social relationships, both within and outside the university, as a key factor in enhancing wellbeing in the academic setting, both for academics and administrative staff. Indeed, academic institutions serve as a bridge between society, the world of work, and the local community.

Finally, the Research Topic addresses the gap between risk assessment and intervention management in academia. In many cases, little attention is paid to how identified risks are managed once they have been assessed. By examining the factors that facilitate the transition from assessment to implementation of interventions in the academic context, Bruno, Dell'Aversana et al. suggest that creating organizational scaffolding and participatory processes in the risk assessment-management pathway can prevent data from being underutilized and increase the chances of implementing interventions to create healthier academic conditions. Without these interventions, the focus on wellbeing in academia risks becoming merely rhetorical, with data rarely influencing management decisions. This could lead to an individualistic approach to wellbeing, placing the burden on individuals to manage their health and stress, while ignoring systemic issues within university structures and programs.

In conclusion, while significant strides have been made in addressing wellbeing in academia, particularly in relation to students, more attention needs to be paid to the working conditions and the quality of life at work of academic staff and technical-administrative staff. The development and implementation of policies that address these challenges are essential for fostering a healthier and more supportive academic environment for all members of the university community.

